# Molecular detection of drug resistant malaria in Southern Thailand

**DOI:** 10.1186/s12936-019-2903-y

**Published:** 2019-08-15

**Authors:** Chaturong Noisang, Christiane Prosser, Wieland Meyer, Waenurama Chemoh, John Ellis, Nongyao Sawangjaroen, Rogan Lee

**Affiliations:** 1 Molecular Mycology Research Laboratory, Centre for Infectious Diseases and Microbiology, Faculty of Medicine and Health, Westmead Clinical School, Marie Bashir Institute for Infectious Diseases and Biosecurity, University of Sydney, Sydney, NSW Australia; 2 Westmead Institute for Medical Research, Westmead, NSW Australia; 3 Westmead Hospital (Research and Education Network), Westmead, NSW Australia; 4 Department of Microbiology, Faculty of Medicine, Princess of Naradhiwas University, Narathiwat, Thailand; 5 School of Life Sciences, University of Technology Sydney, Sydney, NSW Australia; 6 Department of Microbiology, Faculty of Science, Prince of Songkla University, Hat Yai, Songkhla, Thailand; 7 Centre for Infectious Diseases and Microbiology Laboratory Services, ICPMR, Westmead Hospital, Westmead, NSW Australia

**Keywords:** Drug resistance, Molecular surveillance, Southeast Asia, Border malaria, *Plasmodium falciparum*, *Plasmodium vivax*

## Abstract

**Background:**

Drug resistance within the major malaria parasites *Plasmodium vivax* and *Plasmodium falciparum* threatens malaria control and elimination in Southeast Asia. *Plasmodium vivax* first-line treatment drug is chloroquine together with primaquine, and the first-line treatment for *P. falciparum* malaria is artemisinin in combination with a partner drug. *Plasmodium vivax* and *P. falciparum* parasites resistant to their respective first-line therapies are now found within Southeast Asia. The resistance perimeters may include high transmission regions of Southern Thailand which are underrepresented in surveillance efforts.

**Methods:**

This study investigated blood samples from malaria centres in Southern Thailand. Genetic loci associated with drug resistance were amplified and sequenced. Drug resistance associated genes *Pvmdr1*, *Pvcrt*-*o*, *Pvdhfr*, and *Pvdhps* were characterized for 145 cases of *P. vivax* malaria, as well as the artemisinin resistance-associated *Pfkelch13* gene from 91 cases of *P. falciparum* malaria.

**Results:**

*Plasmodium vivax* samples from Southern Thai provinces showed numerous chloroquine and antifolate resistance-associated mutations, including SNP and *Pvcrt*-*o* **K10**-insertion combinations suggestive of chloroquine resistant *P. vivax* phenotypes. A high proportion of the C580**Y** coding mutation (conferring artemisinin resistance) was detected in *P. falciparum* samples originating from Ranong and Yala (where the mutation was previously unreported).

**Conclusions:**

The results demonstrate a risk of chloroquine and antifolate resistant *P. vivax* phenotypes in Southern Thailand, and artemisinin resistant *P. falciparum* observed as far south as the Thai–Malaysian border region. Ongoing surveillance of antimalarial drug resistance markers is called for in Southern Thailand to inform case management.

**Electronic supplementary material:**

The online version of this article (10.1186/s12936-019-2903-y) contains supplementary material, which is available to authorized users.

## Background

*Plasmodium* spp. are quick to adapt due to their high mutation rate, fast genome replication time, and life cycle dynamics [1, 2]. Considering then the massive number of *Plasmodium* organisms in endemic regions, the emergence of de novo resistance would be expected in the presence of drug selective pressure. The major human malaria parasites *Plasmodium vivax* and *Plasmodium falciparum* have historically developed resistance to antimalarial drugs after continued exposure. Population structure may in part explain why *P. falciparum* develops resistance more quickly than *P. vivax*. *Plasmodium vivax* populations are more genetically diverse than *P. falciparum*, even at low transmission intensity, which may make *P. vivax* parasites less susceptible to population bottlenecking (and so slower to develop resistance, as well as being more resilient to malaria interventions) [3, 4]. Antimalarial drug resistance is often observed first in Southeast Asia. Both *Plasmodium* spp. remain endemic, often sympatric, throughout Southeast Asia, though declining *P. falciparum* numbers have resulted in *P. vivax* being the foremost human malaria parasite in the region [5].

*Plasmodium vivax* is a major cause of human malaria in Asia, Central and South America, and Oceania, with an estimated 80 to 400 million cases worldwide each year [5]. According to the World Health Organization (WHO), *P. vivax* malaria cases appear predominantly in Southeast Asia (58%), with a lower number of cases occurring in the Eastern Mediterranean Region (16%) [6]. In Thailand, ~ 6600 people were diagnosed with *P. vivax* infection in 2017 [7]. Malaria control and elimination programmes are underway in response to this, however, these programmes are hindered by drug resistance [8].

Chloroquine-sensitive strains of *P. vivax* are typically cleared from blood within 48 h after patients receive a standard dose of chloroquine (25 mg/kg) [9]. A recurrent parasitaemia between 15 to 35 days after the chloroquine treatment commences is classified as resistant [9, 10]. The first chloroquine resistance in *P. falciparum* was reported from Southeast Asia at the end of the 1950s [8]. After three decades, the first report of chloroquine resistance in *P. vivax* was reported when Australian travelers diagnosed with a *P. vivax* infection returned from Papua New Guinea and failed to eliminate the blood stages by the standard chloroquine treatment [9–12]. Subsequently, chloroquine resistance in *P. vivax* emerged in endemic Asian and South American regions [9–12]. The recommended targets for molecular surveillance of chloroquine resistance in *P. vivax* are a multidrug resistance gene (*Pvmdr1*) and a putative transporter protein gene (*Pvcrt*-*o*) (homologous with *P. falciparum* chloroquine resistance genes) [13, 14].

Although sulfadoxine–pyrimethamine is not recommended to treat *P. vivax* infections, *P. vivax* is exposed to sulfadoxine–pyrimethamine through coinfection with *P. falciparum* (which may be treated by sulfadoxine–pyrimethamine in combination with another partner drug) and by misdiagnosis of *Plasmodium* species. Other drug treatments comprising sulfadoxine–pyrimethamine could place additional selective pressure onto *P. vivax*, for instance the use of antifolate drugs as a malaria chemoprevention for pregnant women (not currently routine practice in Southeast Asia [15]). There have recently been reports of sulfadoxine/pyrimethamine drug resistances associated with genetic mutations [14]. Pyrimethamine resistance was found to be correlated with specific SNPs of dihydrofolate reductase (*Pvdhfr*) which results in decreased enzyme affinity to the drug [14, 16]. Sulfadoxine resistance is found to be associated with SNPs in the dihydropteroate synthetase gene (*Pvdhps*) [14]. In 2017, Nyunt and colleagues reported that ~ 70% of *P. vivax* samples from three study sites contained a quadruple mutation of *Pvdhfr* [14]. The authors concluded that there is likely a high proportion of pyrimethamine resistance genotypes in Myanmar, despite pyrimethamine not being the drug of choice for *P. vivax* infection in the region [14]. Surveillance of resistance is essential for early warning systems and advocacy of appropriate drug policy. Regional data as well as a clear understanding of the mechanisms of *P. vivax* drug resistance are lacking. As *P. falciparum* moves towards elimination in Southeast Asia, it is expected attention will turn to *P. vivax*.

The global first-line treatment for *P. falciparum* malaria is artemisinin-based combination therapy (ACT) [17]. Artemisinin treatment failure was initially observed on the Thai–Cambodian border, and subsequently has spread throughout the greater Mekong subregion of Southeast Asia [18]. Artemisinin resistance is characterized by a parasite clearance half-life of > 5 h, although resistance needs to be considered together with sensitivity to the artemisinin partner drug [19]. Following the first reports of artemisinin resistance in 2008 by Noedl et al, and in 2009 by Dondorp et al. [20, 21], several Single Nucleotide Polymorphisms (SNPs) within the propeller domain of the parasite’s *Pfkelch13* gene have been associated with the resistant phenotype [22]. These *Pfkelch13* SNPs were demonstrated to significantly decrease artemisinin sensitivity when inserted into Cambodian isolates [23]. The *Pfkelch13* coding mutation (C580**Y**) with the strongest association with artemisinin resistance is now found throughout Southeast Asia and is approaching fixation in western Cambodia [22, 24]. Resistance mutations have emerged independently in several Southeast Asian populations [25].

The emergence of artemisinin resistant *P. falciparum* in the greater Mekong subregion is alarming, due to both the absence of alternative first-line therapies and the presence of resistance to ACT partner drugs in the region. The WHO, in response to the threat of an untreatable multi-drug resistant parasite, have implemented a strategy to eliminate *P. falciparum* from the six countries located in the greater Mekong subregion by 2025 [26].

This study aimed to assess the presence of mutations in genes associated with drug resistance in *P. vivax* and *P. falciparum* from southern reaches of the greater Mekong subregion in South Thailand that have not been included in previous studies.

## Methods

### Sample collection

**Fig. 1:**
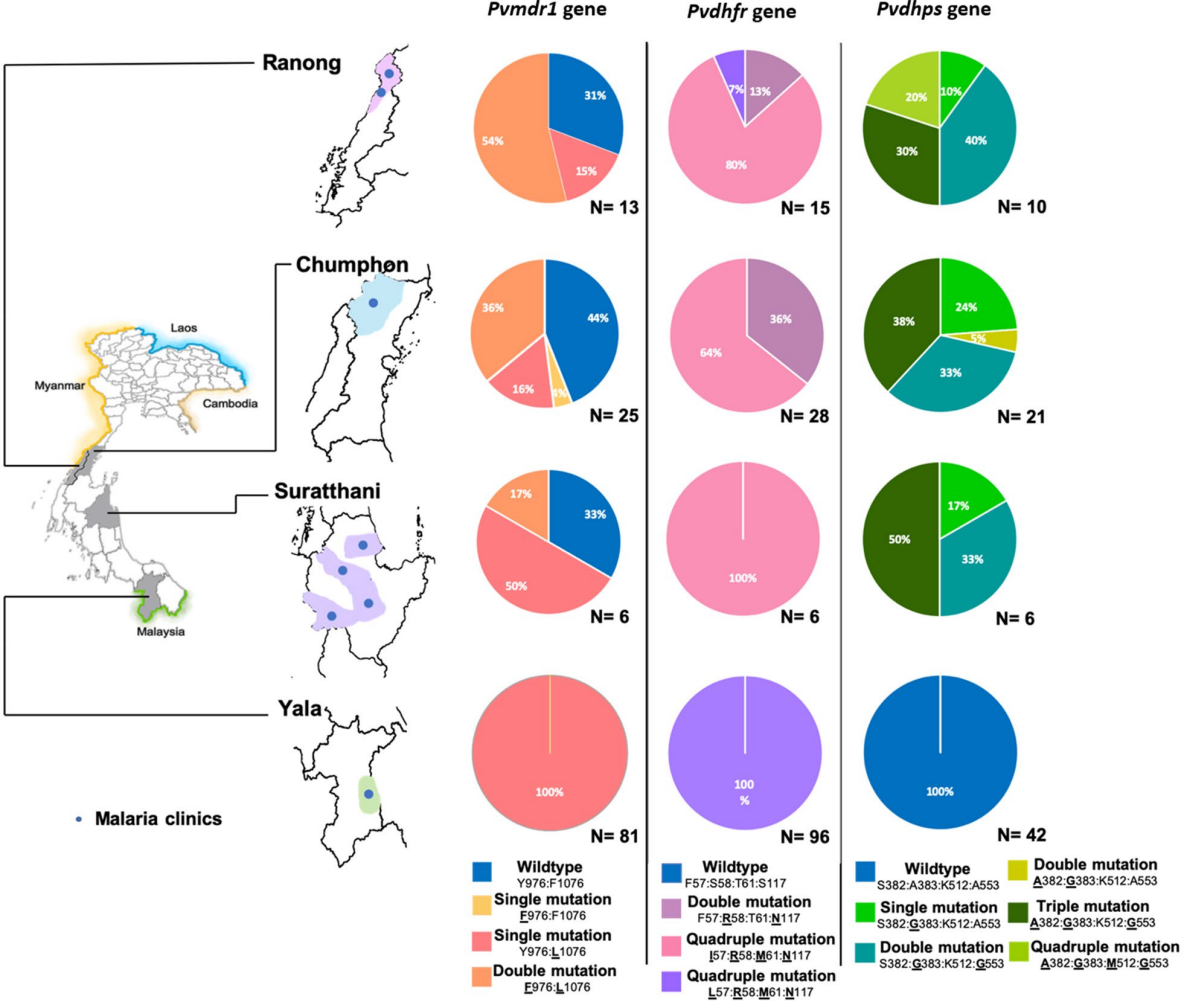
Regional *Plasmodium vivax* genotyping results. Prevalence of point mutations in *Pvmdr1*, *Pvdhfr* and *Pvdhps* genes in *P. vivax* samples from four provinces in Southern Thailand

**Fig. 2:**
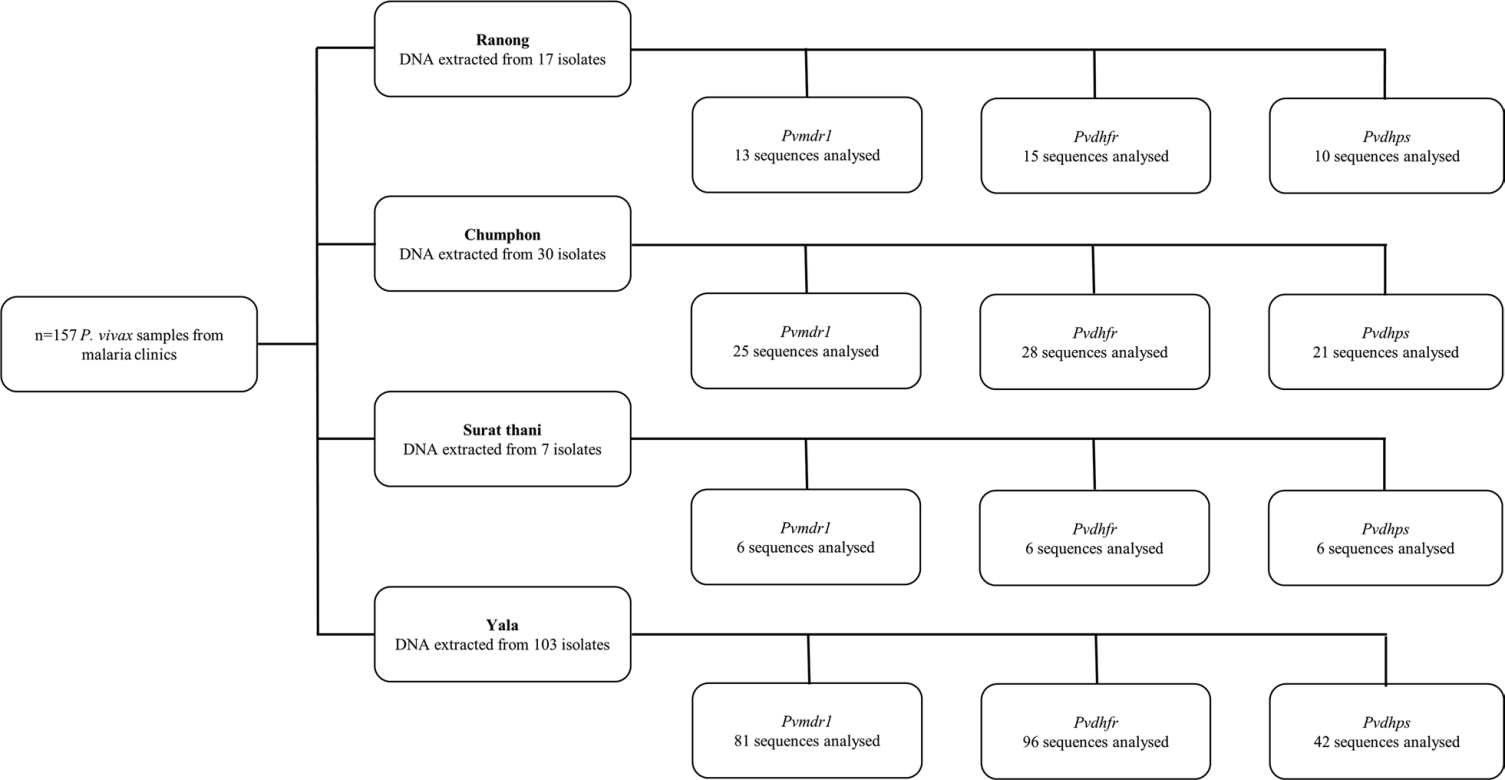
*Plasmodium vivax* sample workflow. Workflow of *P. vivax* samples (n = 157) from four provinces in Southern Thailand

Clinical *P. vivax* and *P. falciparum* samples (n = 157 and n = 91 respectively) were collected from malaria clinics in Southern Thailand (Surat thani, Ranong and Chumphon at Thai–Myanmar border, and Yala at the Thai–Malaysia border, see Figs. 1, 2), over the period 2012 to 2018. Blood samples were obtained by finger-prick, diagnosed by microscopy, and with the parasite species confirmed by Polymerase Chain Reaction (PCR), as described elsewhere [27]. Malaria infected patients included in this study did not present with signs of serious illness and had no previous antimalarial treatment. Two hundred microlitres of whole blood per sample was spotted onto filter paper and sealed for transport. All available patient data was previously collected by patient interview. Accession numbers were assigned to samples, and epidemiologically relevant decoded patient data were recorded (see Additional file 1). All laboratory work was conducted at the Prince of Songkla University, Hat Yai, Songkhla, Thailand.

### DNA extraction

Genomic DNA was extracted from filter paper blood spots using a QIAamp mini DNA kit (QIAGEN, Maryland, USA) as per manufacturer’s directions. DNA quality was confirmed by subjecting DNA to agarose gel electrophoresis. DNA concentrations were measured by spectrophotometric analysis using a Nanodrop® Spectrophotometer ND-1000 at 260 nm and 280 nm. As an experimental control for *P. falciparum*, DNA from laboratory reference strain 3D7 *P. falciparum* kindly provided by Dr Jutta Marfurt (Menzies School of Health Research, NT, Australia) was included. Samples were stored at − 20 °C when not in use.

### *Plasmodium vivax*

A multidrug resistant gene (*Pvmdr1*) and a putative transporter protein gene (*Pvcrt*-*o*) were amplified by PCR using specific oligonucleotide primers (Pvmdr F, Pvmdr R, Pvcrt-o F, and Pvcrt-o R) as shown in Additional file 2. Novel primers were designed to capture the *Pvcrt*-*o* gene: Pvcrt-o F 5′–3′ CAGTGAGAAGCCCCTGTTCG and Pvcrt-o R 5′–3′ CCGCTCATCAGTCTGCAC. A total volume of 50 μl PCR reaction mixture contained 0.2 mM of each dNTP, 1× PCR buffer, 1 μM of each primer, 2.5 mM of MgCl_2_, 2 U of Taq polymerase and 3–5 μl of specimen DNA. PCR cycling conditions were as follows; 95 °C for 5 min [94 °C 30 s, 59 °C 45 s, 72 °C 90 s] × 35 cycles, 72 °C for 7 min.

Nested PCR was used to amplify *Pvdhpf* and *Pvdhps* genes with two sets of oligonucleotide primers (shown in Additional file 2). Nested reactions were carried out in a total volume of 25 μl reaction mixture containing 0.2 mM of each dNTP, 1× PCR buffer, 0.2 μM of each primer, 1.75 mM of MgCl_2_, and 1 U of Taq polymerase. Malaria DNA (3 μl, ~ 2–10 ng/μl human + parasite DNA) was added in outer amplification reactions, and then 0.2 μl of outer reaction product was used as the template for the nested amplification reaction. Nested PCR cycling conditions were as follows: (*Pvdhfr*) 94 °C for 5 min [94 °C 30 s, 58 °C 30 s, 68 °C 45 s] × 40 cycles, 68 °C for 5 min; (*Pvdhps*) 94 °C for 5 min [94 °C 30 s, 59 °C 30 s, 68 °C 45 s] × 40 cycles, 68 °C for 5 min. Other reaction conditions were as per outer reaction.

### *Plasmodium falciparum*

Amplification of the propeller region of the *Pfkelch13* gene was adapted from Kamau et al. 2015 [28]. GeneDB accession number PF3D7_1343700 (http://www.plasmodb.org; accessed 1 March 2019) was used as a reference sequence in these studies, as this curated reference strain is artemisinin susceptible, and contains no *Pfkelch13* mutations. The primers (outer set capturing codons 427–691 of the *Pfkelch13* propeller gene, inner capturing codons 427–676) are listed in Additional file 2.

The PCR reaction conditions were: Primary Master Mix (MM1) of 25 µl total reaction volume containing 2.5 µl 10× PCR buffer (100 mM Tris–HCl, pH 8.3, 500 mM KCl, 15 mM MgCl_2_, 0.01% w/v gelatine), 0.75 µl 50 mM MgCl_2_, 2 µl 2 mM dNTP mix (dinucleotide triphosphates, containing 3 mM dATP and dTTP, 1 mM dCTP and dGTP,), 0.625 µl outer forward primer (10 µM), 0.625 µl outer reverse primer (10 µM), 0.4 µl BioTAQ DNA polymerase (5U/µl) plus 11.1 µl of sterile water. Template DNA = 7 µl/reaction.

The outer PCR product (5 µl) was added to Secondary Master Mix (MM2) containing the same component concentrations of MM1 except that different reverse primer (semi-nested reverse primer Pfk13R) was used. The reaction volume was made up to 50 µl for the semi-nested PCR.

Amplification was carried out in a Sensoquest Labcycler thermal cycler, with cycling conditions for the outer round as follows: initial denaturation at 95 °C for 15 min [95 °C for 1 min, 59 °C for 1 min, 72 °C for 2 min] × 35 cycles, 72 °C for 10 min; semi-nested round: initial denaturation at 95 °C for 15 min [95 °C for 30 s, 60 °C for 1 min, 72 °C for 1 min] × 40 cycles, 72 °C for 10 min.

### SNP analysis

PCR products (amplicons of *Pvdhps*, *Pvmdr1, Pvcrt*-*o, Pvdhpf*, and *Pfkelch13*) were sent to Macrogen (South Korea), for bidirectional sequencing. Sequence data were validated by BLASTN and BLASTX searches (https://blast.ncbi.nlm.nih.gov/Blast.cgi). Sequences were cropped of low-quality ends using 4Peaks (http://nucleobytes.com/4peaks/). Trusted regions of reverse sequences were processed using ReverseComplement (http://www.bioinformatics.org/sms/rev_comp.html). All generated sequences per sample were combined to form a single consensus sequence (contig) using 3cap (http://doua.prabi.fr/software/cap3). Patient contigs were then aligned (using the MEGA7 integrated MUSCLE multiple sequence alignment program) to the reference sequence to identify polymorphisms (Genbank reference no. *Pvdhfr*: XM001615032, *Pvdhps*: XM001617159, *Pvcrt*-*o*: AF314649, and *Pvmdr1*: AY618622, PlasmodDB *Pfkelch13* gene ID PF3D7_1343700). Genetic variants were compared to reported resistance-associated mutations. For the *P. vivax* gene *Pvdhfr:* mutations F57L/I, S58R, T61M, and S117T/N [13]; *Pvdhps* mutations: S382A, A383G, K512M, and A553G [13]; for *Pvcrt*-*o* the K10 insertions (addition of AAG at codon 10) [29]; and for *Pvmdr1*: mutations Y976F and F1076L [13]. Codon 173 of *Pvdhfr* and codon 585 of *Pvdhps* were also analysed (homologs of the *P. falciparum* sulfadoxine–pyrimethamine resistance-associated codons 164 in *Pfdhfr* and 613 in *Pfdhps* respectively [30]). For the *P. falciparum* gene *Pfkelch13*: mutation C580Y [31].

## Results

### Patient data analysis

The patients sampled in this study were mostly young adult men (70% male, with median and mean ages of 32 and 33.7 years old, respectively, and age range of 5 to 86 years). Many infected individuals (80% with known occupation) were at high risk of malaria re-exposure, for instance they worked on coffee or rubber plantations (see Additional file 1).

### *Plasmodium vivax*

#### *Pvmdr1* and *Pvcrt*-*o*

Two point-mutations at codons 976 and 1076 in the *Pvmdr1* gene were identified in 125 isolates from four provinces in Southern Thailand (see Fig. 1). The occurrence of double mutations (Y976**F** and F1076**L**) in *Pvmdr1* was observed in nine Chumphon isolates, seven Ranong isolates, and one Surat thani isolate. A single mutation at codon 976 (Y976**F**) was detected in only one Chumphon isolate, while another single mutation at codon 1076 (F1076**L**) was discovered in 81 Yala isolates, four Chumphon isolates, three Surat thani isolates, and two Ranong isolates. Wildtypes alleles were observed in 11 Chumphon isolates, four Ranong isolates, and two Surat thani isolates. The **K10** insertion (addition of **AAG**) in *Pvcrt*-*o* genes occurred in three of three Ranong isolates only.

#### *Pvdhps*

All mutations at codons 382, 383, 512 and 553 in *Pvdhps* genes were analysed. Quadruple mutations at these codons (S382**A**, A383**G**, K512 **M** and A553**G**) were found in two Ranong isolates. Triple mutations at codons 382, 383, and 553 (**A**382, **G**383 and **G**553) were also detected in eight Chumphon isolates, in three Ranong isolates, and in three Surat thani isolates. Double mutations at codons 383 and 553 (**G**383 and **G**553) appeared in seven Chumphon isolates, four Ranong isolates, and two Surat thani isolates with other double mutations at codons 382 and 383 (**A**382 and **G**383) observed in only one Chumphon isolate. A single mutation at codon 383 (**G**383) occurred in five Chumphon isolates, one Ranong isolate, and one Surat thani isolate, whereas all 42 Yala isolates were found to contain the wildtype allele.

#### *Pvdhfr*

Analysis of polymorphism in *Pvdhfr* genes revealed quadruple mutations at codons 57, 58, 61, and 117 (F57**I**, S58**R**, T61**M** and S117**T**/**N**) in 18 Chumphon isolates, 12 Ranong isolates, and six Surat thani isolates (see Fig. 1). Another combination of quadruple mutations at codons 57, 58, 61, and 117 (**L**57, **R**58, **M**61, and **N**117) were found in 96 Yala isolates and one Ranong isolate. Double mutations at codons 58 and 117 (**R**58 and **N**117) were seen in 10 Chumphon isolates and two Ranong isolates. Both tandem repeat variants in the *Pvdhfr* gene were observed. Type 1 or wildtype sequences, i.e. three repeated sets of four amino acids (5′-**GGDN**-3′) at codons between 88 and 103, were observed in most isolates. Type 2 (deletion sequences including six deleted amino acids at codons 98–103) were observed in 10 Chumphon isolates, two Ranong isolates, and two Yala isolates (see Table 1). All Sequences generated are available on GenBank [Submission ID 2211561]. Sample workflow is shown in Fig. 2.

**Table 1:** Prevalence of tandem repeat variants in the *Pvdhfr* gene in *Plasmodium vivax* samples from four provinces in Southern Thailand

Provinces	*Pvdhfr* gene	
	Type 1 (wildtype)	Type 2 (deletion)
Ranong	13 (86.60%)	2 (13.40%)
Chumphon	18 (64.30%)	10 (35.70%)
Surat thani	6 (100%)	0
Yala	94 (97.90%)	2 (2.10%)

### *Plasmodium falciparum*

#### *Pfkelch13*

**Fig. 3:**
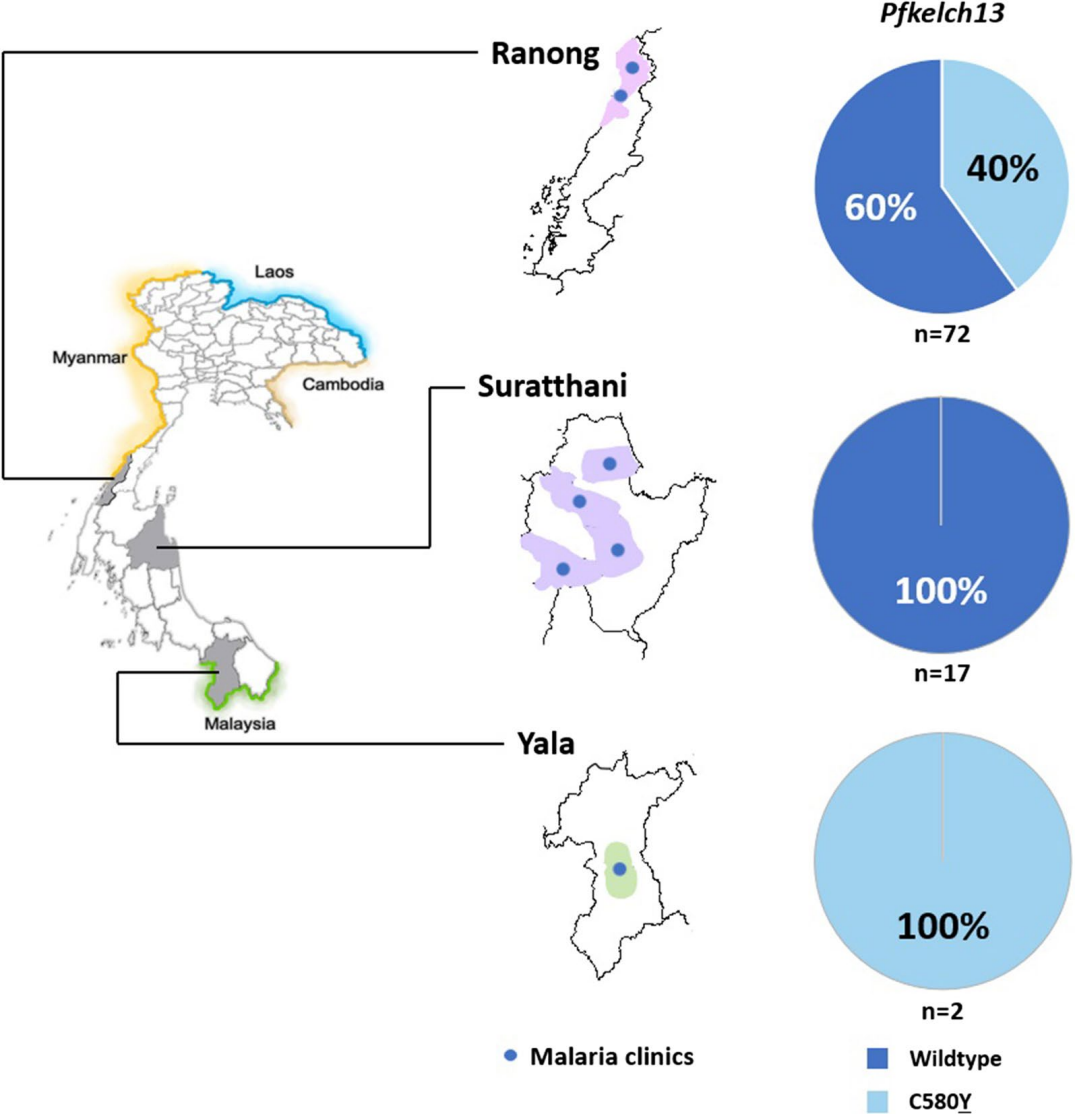
Regional *Plasmodium falciparum* genotyping results. Prevalence of artemisinin-resistance *Pfkelch13* genotypes in *P. falciparum* samples (n = 91) from four provinces in Southern Thailand

60/91 samples aligned identically to the reference *Pfkelch13* gene ID PF3D7_1343700, including all samples originating from Surat Thani. The artemisinin resistance C580**Y** *Pfkelch13* variant was found in 31 samples originating from Ranong and Yala, as detailed in Fig. 3. No other nonsynonymous *Pfkelch13* mutations were observed within the propeller domain region amplified (codons 427 to 676). All sequences are available via GenBank [MK766747–MK766837].

## Discussion

### Malaria control and elimination in Thailand

Malaria control efforts in Thailand have been highly successful, with a greater than 80% reduction in cases over 2007 to 2017 [32, 33]. However, the malaria burden continues to be a public health challenge. The Thai government has stated a national malaria elimination goal of 2024, with the strategy focusing on the detection of asymptomatic malaria together with effective treatment [33, 34].

There are significant challenges to malaria elimination inherent in Thailand’s socioeconomics and geography. Malaria control is complicated in regions bordering other endemic nations by human/vector migration, and as the typically rural settings tend to result in high transmission coupled with limited health services [35]. Similarly, remoteness, limited resources, and the political complexity of border regions often produce suboptimal surveillance responses. The most significant malaria reduction, and most comprehensive surveillance coverage, is limited to central and urban Thai provinces [32]. Thailand’s four international borders now account for 70% of malaria cases [34]. To achieve elimination, upscaled surveillance at Thailand’s peripheries is required, with activities coordinated with neighbouring countries. This includes Thailand’s southern border with Malaysia, where over 30% of the nation’s malaria transmission is now found (the highest parasite prevalence in the country) [34].

### *Plasmodium vivax*

#### *Pvmdr1* and *Pvcrt*-*o* genes polymorphism

Point mutations in *Pvmdr1* and *Pvcrt*-*o* genes are considered strong candidate markers of resistance to the front-line treatment drug chloroquine in *P. vivax,* and their evaluation in molecular resistance surveillance is encouraged. An in vitro drug susceptibility assay became available in 2003, and in 2007 a rise in chloroquine inhibitory concentration (IC_50_) was reported to correlate both with **K10** insertion and with **F**976 mutation of the *Pvmdr1* gene (all Thai isolates carrying the **F**976 mutation showed a 1.7-fold rise in chloroquine IC_50_) [36, 37]. A parallel study in India detected no *Pvmdr1* and *Pvcrt*-*o* gene mutations in chloroquine sensitive *P. vivax* [38]. A study defining the *P. vivax* chloroquine-resistant phenotype in Myanmar by therapeutic efficacy study during 2006–2009 reported a clinical failure after 28 days of treatment in Kawthaung (1.7%) and two further treatment failures in Buthidaung (3.3%). Both provinces were known to have high prevalence of mutant alleles of *Pvmdr1* and *Pvcrt*-*o* [14].

In this study, the *Pvmdr1* gene double mutation (**F**976**L**1076) appeared mainly in Ranong (54%), Chumphon (36%), and Surat thani (17%), close to the Thai–Myanmar border (see Fig. 1). These findings are relatively consistent with previous studies. A study sampling from the border found 49.2% and 18.4% prevalence of the double mutation in 2008 and 2014, respectively [39]. Similarly, a study in Myanmar from 2009 to 2016 found a low proportion of **F**976**L**1076 in Kawthaung (16.7%) and Shwegyin (12.5%) [14]. Findings in this study are somewhat expected, given that Kawthaung (previously surveyed) is adjacent to Ranong and Chumphon, and people frequently travel across the border for work. A 2015 survey of samples from Tak on the Northeast border of Thailand likewise found a significant proportion of the **F**976**L**1076 *Pvmdr1* double mutations (23.3%) [40].

The *Pvmdr1* wildtype (Y976F1076) was observed in this study in moderate proportions in Chumphon (44%), Surat thani (33%), and Ranong (31%) (see Fig. 1). Interestingly, only the *Pvmdr1* wildtype was found in Yala adjacent to the Thai–Malaysian border. Given the consistent genotyping results of other genes studied, it appears that the *P. vivax* population in Yala is homogenous even though this province is not isolated by natural barriers. Although it is located in the far south of Thailand, there is still travel into the region. Yet, the findings of this study suggest that the *P. vivax* haplotypes observed in Ranong, Chumphon and Surat thani have not spread to Yala.

The **K10** *Pvcrt*-*o* gene insertion was found only in Ranong (17.6%) near the Myanmar border, while the wildtype was observed in other Southern Thai provinces. Previous investigations from Myanmar have reported a high proportion of the **K10** insertions in Shwegyin (72.7%), Kawthaung (66.7%), and Buthidaung (48.3%) between 2009 and 2016 [14]. A study as early as 1999 reported significant proportion of the **K10** insertion in samples from Yangon, Myanmar (46.2%), and the insertion was reported in 2008 at a prevalence of 56% on the Northeast Myanmar border in Tak [29]. More recent studies in Thailand, however, did not find the **K10** insertion in either sample from the Thai–Myanmar border area or from the Thai–Cambodia border area [39, 40]. Another 2012 study in central China likewise did not detect the **K10** insertion [41].

The findings of this study suggest that Ranong, which had both the double mutation **F**976**L**107676 in *Pvmdr1* genes, and the **K10** insertion in *Pvcrt*-*o* genes, may have emergent extensively chloroquine resistant *P. vivax* phenotypes. Within Chumphon and Surat thani (where single or double *Pvmdr1* mutations were observed) parasites may have a partial reduction to chloroquine susceptibility, and an increased vulnerability to developing true chloroquine resistance via selection of additional de novo mutation, or via interbreeding with dispersed parasites that carry additional resistance mutations. Conversely, the authors would speculate that chloroquine treatment remains effective in Yala where only one *Pvmdr1* SNP was detected. Limitations of this study include difficulties successfully amplifying the *Pvcrt*-*o* gene from some blood samples. Additionally, the authors were unable to access sample sites within target provinces such as Narathiwat, Pattani and Songkhla. As such, it is possible the **K10** insertion may be present in other provinces not surveyed, or within sites included in this study that were not detected. Further investigation is needed to best inform the *P. vivax* treatment policies for Southern Thai regions.

#### *Pvdhfr* and *Pvdhps* genes polymorphism

Antifolate resistance (pyrimethamine and sulfadoxine) in *P. vivax* is associated with point mutations in *Pvdhfr* and *Pvdhps* genes which are homologous to point mutations in *Pfdhfr* and *Pfdhps* genes in antifolate-resistant *P. falciparum* [12, 14, 39]. In this study, point mutations at codons 57, 58, 61, and 117 in the *Pvdhfr* gene, which is strongly correlated to pyrimethamine resistance, were detected in all sequenced isolates (n = 145). The quadruple mutation (**L**/**I**57**R**58**M**61**N**117) is significantly linked to sulfadoxine–pyrimethamine treatment failure [42] and was the most common genotype observed in the study locations (see Fig. 1). High rates of quadruple *Pvdhfr* mutation **L**/**I**57**R**58**M**61**N**117 (~ 50–80%) and double mutation **R**58**N**117 (~ 50%) have been reported over the past decade from the Thai–Myanmar border [14, 39]. These observations are consistent with this study’s finding of the double mutation (**R**58**N**117) in Chumphon (36%), and Ranong (13%). The wildtype genotype was not observed in this study.

Tandem repeat variants within the *Pvdhfr* gene are also suggested to be associated with *P. vivax* antifolate resistance [43]. In this study, the highest frequency of the tandem repeat variants was Type 1 (wildtype) which were observed in Surat thani (100%), Yala (97.9%), Ranong (86.6%), and Chumphon (64.3%). This is consistent with a previous study reporting most Thai–Myanmar border isolates as Type 1, along with a single mutation at codon 117 (**N**117) [44, 45]. Type 2 (deletion) was found in Chumphon (35.7%), Ranong (13.4%), and Yala (2.1%). The role of tandem repeat variants in antifolate resistant *P. vivax* remains not fully understood.

Point mutations at codons 382, 383, 512, and 553 in *Pvdhps* gene are linked to sulfadoxine resistance in *P. vivax* [14, 39, 46]. In this study, *Pvdhps* gene polymorphisms were found to vary across Southern Thailand. This study detected alleles consistent with the pattern of *Pvdhps* gene polymorphisms reported by a previous study on the Thai–Myanmar border in 2014 [39]. Furthermore, in the past decade a high rate (~ 75%) of quadruple mutation **A**382**G**383**M**512**G**553 has been reported for Shywegyin and Kawthaung in Myanmar [14]. Proceeding this study, there was no data available for other southern regions of Thailand and along the Thai–Malaysia border. The outcomes of this study suggest the genotypes previously reported in other areas of Thailand and Myanmar have spread to southern provinces including Ranong Chumphon, and Surat thani, whereas it appears that the Yala parasite populations retain the wildtype *Pvdhps* gene which has disappeared in other parts of Thailand.

Genotypes detected in this study containing the quadruple mutation alleles of both the *Pvdhfr* and *Pvdhps* genes may be associated with sulfadoxine–pyrimethamine resistance in Ranong, Chumphon, and Surat thani (within the Thai–Myanmar border region). The co-infection of *P. falciparum* and *P. vivax* is common in Thailand [47, 48], where sulfadoxine–pyrimethamine treatment was the first-line drug against *P. falciparum* until 1996, and currently remains used indiscriminately to treat fever in self-care situations [39, 45]. Other causes of *P. vivax* sulfadoxine–pyrimethamine exposure are the presumptive treatment of falciparum malaria without laboratory confirmation and erroneous diagnosis of *Plasmodium* spp. [49]. The quadruple mutation *Pvdhfr* allele was only detected in Yala. One reason for this may be the lower sulfadoxine–pyrimethamine drug pressure *P. vivax* parasites are subjected to in Yala compared to neighbouring provinces. However, *P. vivax* is known to be the dominant species in this area.

### Plasmodium falciparum

#### *Pfkelch13* gene polymorphism

The presence of C580**Y** in *P. falciparum* populations in Ranong and in Southern Thai provinces near the Myanmar border is expected given previous reports on the spread of resistance to the front-line therapy drug artemisinin in parasites in Thailand over the past 5 years [50]. Clinical investigation by the Tracking Resistance to Artemisinins Collaboration (TRAC) and molecular investigation conducted by the MalariaGEN *Plasmodium falciparum* project do not extend to Southern regions on the Malaysian border, and to the authors’ knowledge the artemisinin resistance genotypes observed throughout the greater Mekong subregion have not been reported in Yala [19, 51]. The lack of molecular surveillance in far Southern districts may be due to the operational difficulties of the area, combined with a very low transmission rate of *P. falciparum*.

The *PfPailin* lineage (which contain both the C580**Y** *Pfkelch13*-mediated artemisinin resistance, and resistance to the partner drug piperaquine) has migrated from its origin in western Cambodia along the Cambodian borders of Thailand and Laos [52]. In 2017, *PfPailin* parasites were observed to be causing treatment failure as far as Binh Phuoc in southern Vietnam [53]. In response to the threat of an untreatable multi-drug resistant parasite, the WHO strategy for malaria elimination in the greater Mekong subregion plans to eliminate *P. falciparum* from the six countries by 2025 [26]. Although in recent years transmission of *P. falciparum* in South Thailand has significantly declined (to less than one case per thousand population for most Southern provinces, and zero to one case per thousand population for provinces bordering Malaysia) the finding of C580**Y** *Pfkelch13 P. falciparum* in Yala is noteworthy [5]. The resistance genotype represents a risk of sustaining a reservoir of artemisinin resistance in Southern Thailand, which could frustrate elimination plans. *Pfkelch13* resistance genotypes are shown to have both disseminated throughout the greater Mekong subregion, and to have repeatedly independently emerged [25]. While it is most likely the case that the C580**Y** genotypes observed in Yala migrated south from resistance-endemic regions of Thailand, there is a possibility of de novo *Pfkelch13* mutation [54]. The authors recommend awareness and on-going surveillance of artemisinin resistant *P. falciparum* not only for Southern Thailand, but for peninsular Malaysia and Sumatera where there is a risk of dissemination.

### Additional considerations

The convenience sampling in this study (collecting from febrile patients who had presented to accessible malaria centres) creates limitations. Many sites did not have a sample size adequate to represent the true prevalence of resistance alleles (particularly *P. falciparum* collection from the Thai–Malaysian border). Molecular findings such as those in this study can serve to alert of the presence of resistance alleles within regions with little or no reported molecular data. However, ongoing and comprehensive molecular surveillance is required to inform policymakers and to enable the stated Thai malaria elimination strategy.

Patient follow up is restricted and so treatment outcomes for patients carrying *P. vivax* and *P. falciparum* with resistance-associated genotypes were not available. The re-exposure risk inherent in the typical patient’s occupation (see Additional file 1) could additionally lead to masking of treatment failure. The authors recommend additional molecular surveillance of both *P. vivax* and *P. falciparum* drug resistance markers in Southern Thailand. Gathering surveillance data linked with treatment outcomes, and more extensive epidemiologically relevant patient information, would additionally be a valuable opportunity to improve the understanding of the genetic basis of *P. vivax* resistance.

## Conclusions

There is an urgent need for drug resistance surveillance of *P. vivax* and *P. falciparum* in the rural and border areas of Southern Thailand. Given the circumstances discussed in this paper (masking of early treatment failure, inherent resource limitations, and sociopolitical barriers to malaria control in these regions) this might be best achieved with ongoing molecular surveillance which extends to the level of community malaria centres. Failing to achieve comprehensive coverage of this significant region would be detrimental to the Thai and Southeast Asian strategies for malaria elimination.

## Additional files

**Additional file 1: Table S1.** A patient information and results summary (see Table B for *P. vivax* results summary by specimen).

**Additional file 2: Table S2.** PCR primers used in study.

## Data Availability

Deidentified patient data generated is included in this published article (and its additional files). Further patient datasets generated are not publicly available due to patient privacy. All generated sequences are available in GenBank [*P. falciparum* sequences MK766747–MK766837, and *P. vivax* sequences—*submission ID 2211561*-].

## Abbreviations

ACT: artemisinin-based combination therapy
PCR: Polymerase Chain Reaction
SNP: Single Nucleotide Polymorphism
TRAC: Tracking Resistance to Artemisinins Collaboration
WHO: World Health Organization
