# Supplementary material for: Molecular detection of drug resistant malaria in Southern Thailand
Source: Malar J. 2019 Aug 15;18:275. doi: 10.1186/s12936-019-2903-y (PMC6694568; doi:10.1186/s12936-019-2903-y)
Supplement: Supplementary file 1 — Additional file 1: Table S1. A patient information and results summary (see Table B for P. vivax results summary by specimen). [file 12936_2019_2903_MOESM1_ESM.docx]

**Additional file 1: Table S1.** A Patient information and results summary (see Table B for *P. vivax* results summary by specimen).

| **Specimen ID** | **Nationality** | **Age** | **Sex** | **Occupation** | **Location** | **Collection** | **Spp.** |  |
| --- | --- | --- | --- | --- | --- | --- | --- | --- |
|  |  |  |  |  |  |  |  |  |
| **T1** | Burmese | 27 | M | Ruber Plantation | Ranong | 2012 | *P. v* |  |
| **T2** | Thai | 35 | F | Coffee Plantation | Ranong | 2012 | *P. v* |  |
| **T3** | Thai | 36 | M | Coffee Plantation | Ranong | 2012 | *P. v* |  |
| **T4** | Burmese | 16 | M | Ruber Plantation | Ranong | 2012 | *P. v* |  |
| **T5** | Burmese | 20 | M | Ruber Plantation | Ranong | 2012 | *P. v* |  |
| **T6** | Thai | 26 | M | Ruber Plantation | Ranong | 2012 | *P. v* |  |
| **T7** | Thai | 81 | M | Ruber Plantation | Ranong | 2015 | *P. v* |  |
| **T8** | Burmese | 20 | M | Ruber Plantation | Ranong | 2015 | *P. v* |  |
| **T9** | Thai | 14 | M |  | Ranong | 2015 | *P. v* |  |
| **T10** | Thai | 38 | M | Ruber Plantation | Ranong | 2015 | *P. v* |  |
| **T11** | Burmese | 19 | M | Ruber Plantation | Ranong | 2015 | *P. v* |  |
| **T12** | Thai | 22 | M | Military | Ranong | 2015 | *P. v* |  |
| **T13** | Burmese | 25 | M | Ruber Plantation | Ranong | 2015 | *P. v* |  |
| **T14** | Thai | 54 |  | Ruber Plantation | Pangnga | 2012 | *P. v* |  |
| **T15** | Thai | 58 | M | Labor | Chumphon | 2012 | *P. v* |  |
| **T16** | Thai | 42 | M | Ruber Plantation | Surat thani | 2012 | *P. v* |  |
| **T17** | Burmese | 18 | M | Ruber Plantation | Pangnga | 2012 | *P. v* |  |
| **T18** | Thai | 38 | M | Ruber Plantation | Pangnga | 2012 | *P. v* |  |
| **T19** | Thai | 44 | M | Ruber Plantation | Surat thani | 2014 | *P. v* |  |
| **T20** | Thai | 42 | F | Ruber Plantation | Ranong | 2014 | *P. v* |  |
| **T21** | Burmese | 38 | M | Ruber Plantation | Ranong | 2014 | *P. v* |  |
| **T22** | Thai | 53 |  |  | Ranong | 2014 | *P. v* |  |
| **T23** | Thai | 63 | F | Ruber Plantation | Ranong | 2014 | *P. v* |  |
| **T24** | Thai | 24 | M | Labor | Ranong | 2014 | *P. v* |  |
| **T25** | Thai | 53 | M | Ruber Plantation | Ranong | 2014 | *P. v* |  |
| **T26** | Burmese | 18 | F | Ruber Plantation | Ranong | 2014 | *P. v* |  |
| **T27** | Burmese | 15 | M | Ruber Plantation | Ranong | 2014 | *P. v* |  |
| **T28** | Burmese | 12 | M | Ruber Plantation | Ranong | 2014 | *P. v* |  |
| **T29** | Burmese | 15 | M | Ruber Plantation | Ranong | 2014 | *P. v* |  |
| **T30** | Burmese | 32 | M | Ruber Plantation | Ranong | 2014 | *P. v* |  |
| **T31** | Burmese | 38 | F | Ruber Plantation | Ranong | 2014 | *P. v* |  |
| **T32** | Thai | 50 | F | Ruber Plantation | Chumphon | 2014 | *P. v* |  |
| **T33** | Thai | 21 | M | Palm garden | Chumphon | 2014 | *P. v* |  |
| **T34** | Thai | 86 | F | Ruber Plantation | Prchuap khiri khan | 2014 | *P. v* |  |
| **T35** | Thai | 46 | M | Palm garden | Chumphon | 2014 | *P. v* |  |
| **T36** | Thai | 36 | F | Coffee Plantation | Chumphon | 2014 | *P. v* |  |
| **T37** | Thai | 46 | M | Ruber Plantation | Chumphon | 2014 | *P. v* |  |
| **T38** | Thai | 38 | M | Labor | Chumphon | 2014 | *P. v* |  |
| **T39** | Thai | 23 | M | Ruber Plantation | Chumphon | 2014 | *P. v* |  |
| **T40** | Thai | 40 | M | Ruber Plantation | Prchuap khiri khan | 2014 | *P. v* |  |
| **T41** | Thai | 30 | F | Ruber Plantation | Chumphon | 2014 | *P. v* |  |
| **T42** | Thai | 30 | M | Ruber Plantation | Surat thani | 2014 | *P. v* |  |
| **T43** | Thai | 43 | M | Ruber Plantation | Surat thani | 2014 | *P. v* |  |
| **T44** | Thai | 32 | M | Coffee Plantation | Chumphon | 2014 | *P. v* |  |
| **T45** | Loas | 20 | M | Labor | Chumphon | 2014 | *P. v* |  |
| **T46** | Thai | 24 | F | Ruber Plantation | Chumphon | 2014 | *P. v* |  |
| **T47** | Thai | 15 | M | Ruber Plantation | Chumphon | 2014 | *P. v* |  |
| **T48** | Thai | 8 | M | Student | Chumphon | 2014 | *P. v* |  |
| **T49** | Burmese | 30 | M | Ruber Plantation | Chumphon | 2014 | *P. v* |  |
| **T50** | Thai | 54 | F | Ruber Plantation | Chumphon | 2014 | *P. v* |  |
| **T51** | Thai | 45 | F | Labor | Chumphon | 2014 | *P. v* |  |
| **T52** | Thai | 54 | M | Military | Chumphon | 2014 | *P. v* |  |
| **T53** | Thai | 31 | M | Office | Chumphon | 2014 | *P. v* |  |
| **T54** | Thai | 45 | F | Ruber Plantation | Chumphon | 2014 | *P. v* |  |
| **T55** | Thai | 14 | M | Student | Chumphon | 2014 | *P. v* |  |
| **T56** | Thai | 47 | M | Ruber Plantation | Chumphon | 2014 | *P. v* |  |
| **T57** | Thai | 28 | F | Ruber Plantation | Chumphon | 2014 | *P. v* |  |
| **T58** | Thai | 47 | M | Ruber Plantation | Chumphon | 2014 | *P. v* |  |
| **T59** | Burmese | 27 | M | Labor | Chumphon | 2014 | *P. v* |  |
| **T60** | Burmese | 25 | F | Ruber Plantation | Chumphon | 2014 | *P. v* |  |
| **T61** | Thai | 30 | M | Fruit garden | Chumphon | 2014 | *P. v* |  |
| **T62** | Burmese | 30 | M | Ruber Plantation | Chumphon | 2014 | *P. v* |  |
| **T63** | Thai | 22 | M | Business owner | Chumphon | 2014 | *P. v* |  |
| **T64** | Thai | 30 | F | Housewife | Chumphon | 2014 | *P. v* |  |
| **T65** | Thai | 5 | M |  | Chumphon | 2014 | *P. v* |  |
| **T66** | Thai | 37 |  | Ruber Plantation | Surat thani | 2014 | *P. v* |  |
| **T67** | Thai | 9 | F |  | Surat thani | 2014 | *P. v* |  |
| **T68** | Thai | 35 | M |  | Surat thani | 2014 | *P. v* |  |
| **T69** | Thai | 33 | F | Ruber Plantation | Surat thani | 2014 | *P. v* |  |
| **T70** | Thai | 23 |  | Ruber Plantation | Surat thani | 2014 | *P. v* |  |
| **T71** | Thai | 52 | M | Labor | Surat thani | 2014 | *P. v* |  |
| **T72** | Burmese | 30 | M | Ruber Plantation | Surat thani | 2014 | *P. v* |  |
| **T73** | Thai | 15 | F | Student | Surat thani | 2014 | *P. v* |  |
| **T74** | Thai | 39 | M | Labor | Ranong | 2012 | *P. v* |  |
| **T75** | Burmese | 15 | M | Labor | Ranong | 2012 | *P. v* |  |
| **T76** | Burmese | 27 | M | Ruber Plantation | Ranong | 2012 | *P. v* |  |
| **T77** | Thai | 45 | M | Labor | Surat thani | 2012 | *P. v* |  |
| **T78** | Burmese | 24 | F | Ruber Plantation | Ranong | 2012 | *P. v* |  |
| **T79** | Thai | 25 | F | Ruber Plantation | Chumphon | 2012 | *P. v* |  |
| **T80** | Burmese | 5 | M |  | Chumphon | 2012 | *P. v* |  |
| **T81** | Burmese | 14 | M |  | Chumphon | 2012 | *P. v* |  |
| **T82** | Burmese | 5 | F |  | Chumphon | 2012 | *P. v* |  |
| **T83** | Burmese | 33 | F |  | Chumphon | 2012 | *P. v* |  |
| **T84** | Burmese | 14 | M |  | Ranong | 2012 | *P. v* |  |
| **T85** | Burmese | 13 | F |  | Ranong | 2012 | *P. v* |  |
| **T86** | Burmese | 35 | M | Ruber Plantation | Chumphon | 2012 | *P. v* |  |
| **T87** | Burmese | 5 | M |  | Ranong | 2012 | *P. v* |  |
| **T88** | Thai | 55 | M | Coffee Plantation | Ranong | 2012 | *P. v* |  |
| **T89** | Burmese | 25 | M | Ruber Plantation | Pangnga | 2012 | *P. v* |  |
| **T90** | Thai | 60 | M | Ruber Plantation | Chumphon | 2012 | *P. v* |  |
| **T91** | Thai | 49 | M | Palm garden | Chumphon | 2012 | *P. v* |  |
| **T92** | Burmese | 46 | M | Ruber Plantation | Chumphon | 2012 | *P. v* |  |
| **T93** | Thai | 69 | F | Ruber Plantation | Surat thani | 2012 | *P. v* |  |
| **T94** | Thai |  |  |  | Yala | 2017 | *P. v* |  |
| **T95** | Thai |  |  |  | Yala | 2017 | *P. v* |  |
| **T96** | Thai |  |  |  | Yala | 2017 | *P. v* |  |
| **T97** | Thai |  |  |  | Yala | 2017 | *P. v* |  |
| **T98** | Thai | 50 | F | Ruber Plantation | Yala | 2017 | *P. v* |  |
| **T99** | Thai | 30 | F | Ruber Plantation | Yala | 2017 | *P. v* |  |
| **T100** | Thai | 43 | F | Ruber Plantation | Yala | 2017 | *P. v* |  |
| **T101** | Thai | 30 | M | Ruber Plantation | Yala | 2017 | *P. v* |  |
| **T102** | Thai | 40 | F | Ruber Plantation | Yala | 2017 | *P. v* |  |
| **T103** | Thai | 39 | M | Ruber Plantation | Yala | 2017 | *P. v* |  |
| **T104** | Thai | 13 | F | Ruber Plantation | Yala | 2017 | *P. v* |  |
| **T105** | Thai | 27 | M | Ruber Plantation | Yala | 2017 | *P. v* |  |
| **T106** | Thai | 31 | M | Ruber Plantation | Yala | 2017 | *P. v* |  |
| **T107** | Thai | 34 | M | Ruber Plantation | Yala | 2017 | *P. v* |  |
| **T108** | Thai | 17 | M | Student | Yala | 2017 | *P. v* |  |
| **T109** | Thai | 26 | M | Ruber Plantation | Yala | 2017 | *P. v* |  |
| **T110** | Thai | 57 | M | Ruber Plantation | Yala | 2017 | *P. v* |  |
| **T111** | Thai | 31 | F | Ruber Plantation | Yala | 2017 | *P. v* |  |
| **T112** | Thai | 34 | M | Ruber Plantation | Yala | 2017 | *P. v* |  |
| **T113** | Thai | 47 | M | Ruber Plantation | Yala | 2017 | *P. v* |  |
| **T114** | Thai | 30 | M | Ruber Plantation | Yala | 2017 | *P. v* |  |
| **T115** | Thai | 52 | M | Ruber Plantation | Yala | 2017 | *P. v* |  |
| **T116** | Thai | 41 | M | Ruber Plantation | Yala | 2017 | *P. v* |  |
| **T117** | Thai | 55 | M | Ruber Plantation | Yala | 2017 | *P. v* |  |
| **T118** | Thai | 29 | M | Military | Yala | 2017 | *P. v* |  |
| **T119** | Thai | 26 | F | Ruber Plantation | Yala | 2017 | *P. v* |  |
| **T120** | Thai | 14 | F | Student | Yala | 2017 | *P. v* |  |
| **T121** | Thai | 56 | M | Ruber Plantation | Yala | 2017 | *P. v* |  |
| **T122** | Thai | 34 | M | Ruber Plantation | Yala | 2017 | *P. v* |  |
| **T123** | Thai | 18 | F | Student | Yala | 2017 | *P. v* |  |
| **T124** | Thai | 36 | M | Ruber Plantation | Yala | 2017 | *P. v* |  |
| **T125** | Thai | 35 | M | Ruber Plantation | Yala | 2017 | *P. v* |  |
| **T126** | Thai | 25 | M | Student | Yala | 2017 | *P. v* |  |
| **T127** | Thai | 27 | F | Ruber Plantation | Yala | 2017 | *P. v* |  |
| **T128** | Thai | 64 | M | Ruber Plantation | Yala | 2017 | *P. v* |  |
| **T129** | Thai | 34 | M | Ruber Plantation | Yala | 2017 | *P. v* |  |
| **T130** | Thai | 35 | F | Ruber Plantation | Yala | 2017 | *P. v* |  |
| **T131** | Thai | 12 | F | Student | Yala | 2017 | *P. v* |  |
| **T132** | Thai | 56 | M | Ruber Plantation | Yala | 2017 | *P. v* |  |
| **T133** | Thai | 45 | F | Ruber Plantation | Yala | 2017 | *P. v* |  |
| **T134** | Thai | 62 | F | Ruber Plantation | Yala | 2017 | *P. v* |  |
| **T135** | Thai | 38 | M | Ruber Plantation | Yala | 2017 | *P. v* |  |
| **T136** | Thai | 20 | M | Student | Yala | 2017 | *P. v* |  |
| **T137** | Thai | 25 | F | Student | Yala | 2017 | *P. v* |  |
| **T138** | Thai | 27 | M | Ruber Plantation | Yala | 2017 | *P. v* |  |
| **T139** | Thai | 15 | M | Student | Yala | 2017 | *P. v* |  |
| **T140** | Thai | 17 | M | Student | Yala | 2017 | *P. v* |  |
| **T141** | Thai | 15 | F | Student | Yala | 2017 | *P. v* |  |
| **T142** | Thai | 46 | M | Ruber Plantation | Yala | 2017 | *P. v* |  |
| **T143** | Thai | 51 | F | Ruber Plantation | Yala | 2017 | *P. v* |  |
| **T144** | Thai |  |  |  | Yala | 2017 | *P. v* |  |
| **T145** | Thai |  |  |  | Yala | 2017 | *P. v* |  |
| **T146** | Thai |  |  |  | Yala | 2017 | *P. v* |  |
| **T147** | Thai |  |  |  | Yala | 2017 | *P. v* |  |
| **T148** |  |  |  |  | Yala | 2018 | *P. v* |  |
| **T149** |  |  |  |  | Yala | 2018 | *P. v* |  |
| **T150** |  |  |  |  | Yala | 2018 | *P. v* |  |
| **T151** |  |  |  |  | Yala | 2018 | *P. v* |  |
| **T152** |  |  |  |  | Yala | 2018 | *P. v* |  |
| **T153** |  |  |  |  | Yala | 2018 | *P. v* |  |
| **T154** |  |  |  |  | Yala | 2018 | *P. v* |  |
| **T155** |  |  |  |  | Yala | 2018 | *P. v* |  |
| **T156** |  |  |  |  | Yala | 2018 | *P. v* |  |
| **T157** |  |  |  |  | Yala | 2018 | *P. v* |  |
| **T158** |  |  |  |  | Yala | 2018 | *P. v* |  |
| **T159** |  |  |  |  | Yala | 2018 | *P. v* |  |
| **T160** |  |  |  |  | Yala | 2018 | *P. v* |  |
| **T161** |  |  |  |  | Yala | 2018 | *P. v* |  |
| **T162** |  |  |  |  | Yala | 2018 | *P. v* |  |
| **T163** |  |  |  |  | Yala | 2018 | *P. v* |  |
| **T164** |  |  |  |  | Yala | 2018 | *P. v* |  |
| **T165** |  |  |  |  | Yala | 2018 | *P. v* |  |
| **T166** |  |  |  |  | Yala | 2018 | *P. v* |  |
| **T167** |  |  |  |  | Yala | 2018 | *P. v* |  |
| **T168** |  |  |  |  | Yala | 2018 | *P. v* |  |
| **T169** |  |  |  |  | Yala | 2018 | *P. v* |  |
| **T170** |  |  |  |  | Yala | 2018 | *P. v* |  |
| **T171** |  |  |  |  | Yala | 2018 | *P. v* |  |
| **T172** |  |  |  |  | Yala | 2018 | *P. v* |  |
| **T173** |  |  |  |  | Yala | 2018 | *P. v* |  |
| **T174** |  |  |  |  | Yala | 2018 | *P. v* |  |
| **T175** |  |  |  |  | Yala | 2018 | *P. v* |  |
| **T176** |  |  |  |  | Yala | 2018 | *P. v* |  |
| **T177** |  |  |  |  | Yala | 2018 | *P. v* |  |
| **T178** |  |  |  |  | Yala | 2018 | *P. v* |  |
| **T179** |  |  |  |  | Yala | 2018 | *P. v* |  |
| **T180** |  |  |  |  | Yala | 2018 | *P. v* |  |
| **T181** |  |  |  |  | Yala | 2018 | *P. v* |  |
| **T182** |  |  |  |  | Yala | 2018 | *P. v* |  |
| **T183** |  |  |  |  | Yala | 2018 | *P. v* |  |
| **T184** |  |  |  |  | Yala | 2018 | *P. v* |  |
| **T185** |  |  |  |  | Yala | 2018 | *P. v* |  |
| **T186** |  |  |  |  | Yala | 2018 | *P. v* |  |
| **T187** |  |  |  |  | Yala | 2018 | *P. v* |  |
| **T188** |  |  |  |  | Yala | 2018 | *P. v* |  |
| **T189** |  |  |  |  | Yala | 2018 | *P. v* |  |
| **T190** |  |  |  |  | Yala | 2018 | *P. v* |  |
| **T191** |  |  |  |  | Yala | 2018 | *P. v* |  |
| **T192** |  |  |  |  | Yala | 2018 | *P. v* |  |
| **T193** |  |  |  |  | Yala | 2018 | *P. v* |  |
| **T194** |  |  |  |  | Yala | 2018 | *P. v* |  |
| **T195** |  |  |  |  | Yala | 2018 | *P. v* |  |
| **T196** |  |  |  |  | Yala | 2018 | *P. v* |  |
| **T197** |  |  |  |  | Yala | 2018 | *P. v* |  |
| **Isolate** | **Nationality** | **Age** | **Sex** | **Occupation** | **Location** | **Collection** | **Spp.** | **Result** |
| **H1** | Thai | 31 | F | Ruber plantation | Surat thani | 2014 | *P. f* | wt |
| **H2** | Thai | 37 | M | Ruber plantation | Ranong | 2012 | *P. f* | C580Y |
| **H3** | Thai | 47 | M | Ruber plantation | Surat thani | 2014 | *P. f* | wt |
| **H4** | Thai | 42 | M | Ruber plantation | Surat thani | 2014 | *P. f* | wt |
| **H5** | Burmese | 30 | M | Ruber plantation | Ranong | 2014 | *P. f* | C580Y |
| **H6** | Thai | 40 | M | Ruber plantation | Surat thani | 2014 | *P. f* | wt |
| **H7** | Thai | 39 | M | Ruber plantation | Surat thani | 2014 | *P. f* | wt |
| **H8** | Thai | 30 | M | Ruber plantation | Surat thani | 2014 | *P. f* | wt |
| **H9** | Thai | 47 | M | Ruber plantation | Surat thani | 2014 | *P. f* | wt |
| **H10** | Thai | 26 | F | Ruber plantation | Surat thani | 2014 | *P. f* | wt |
| **H11** | Thai | 36 | F | Ruber plantation | Surat thani | 2014 | *P. f* | wt |
| **H12** | Thai | 40 | M | Ruber plantation | Surat thani | 2014 | *P. f* | wt |
| **H13** | Thai | 40 | M | Ruber plantation | Surat thani | 2014 | *P. f* | wt |
| **H14** | Thai | 33 | F | Ruber plantation | Surat thani | 2014 | *P. f* | wt |
| **H15** | Thai | 30 | F | Ruber plantation | Surat thani | 2014 | *P. f* | wt |
| **H16** | Thai | 22 | F | Ruber plantation | Surat thani | 2014 | *P. f* | wt |
| **H17** | Thai | 67 | F | Ruber plantation | Surat thani | 2014 | *P. f* | wt |
| **H18** | Thai | 54 | F | Ruber plantation | Surat thani | 2014 | *P. f* | wt |
| **H19** | Thai | 42 | M | Ruber plantation | Surat thani | 2014 | *P. f* | wt |
| **H20** |  |  |  |  | Ranong | 2015 | *P. f* | C580Y |
| **H21** |  |  |  |  | Ranong | 2015 | *P. f* | C580Y |
| **H22** |  |  |  |  | Ranong | 2015 | *P. f* | wt |
| **H23** |  |  |  |  | Ranong | 2015 | *P. f* | C580Y |
| **H24** |  |  |  |  | Ranong | 2015 | *P. f* | C580Y |
| **H25** |  |  |  |  | Ranong | 2015 | *P. f* | C580Y |
| **H26** |  |  |  |  | Ranong | 2015 | *P. f* | C580Y |
| **H27** |  |  |  |  | Ranong | 2015 | *P. f* | wt |
| **H28** |  |  |  |  | Ranong | 2015 | *P. f* | C580Y |
| **H29** |  |  |  |  | Ranong | 2015 | *P. f* | wt |
| **H30** |  |  |  |  | Ranong | 2015 | *P. f* | wt |
| **H31** |  |  |  |  | Ranong | 2015 | *P. f* | wt |
| **H32** |  |  |  |  | Ranong | 2015 | *P. f* | wt |
| **H33** |  |  |  |  | Ranong | 2015 | *P. f* | wt |
| **H34** |  |  |  |  | Ranong | 2015 | *P. f* | wt |
| **H35** |  |  |  |  | Ranong | 2015 | *P. f* | C580Y |
| **H36** |  |  |  |  | Ranong | 2015 | *P. f* | wt |
| **H37** |  |  |  |  | Ranong | 2015 | *P. f* | wt |
| **H38** |  |  |  |  | Ranong | 2015 | *P. f* | wt |
| **H39** |  |  |  |  | Ranong | 2015 | *P. f* | wt |
| **H40** |  |  |  |  | Ranong | 2015 | *P. f* | C580Y |
| **H41** |  |  |  |  | Ranong | 2015 | *P. f* | C580Y |
| **H42** |  |  |  |  | Ranong | 2015 | *P. f* | C580Y |
| **H43** |  |  |  |  | Ranong | 2015 | *P. f* | C580Y |
| **H44** |  |  |  |  | Ranong | 2015 | *P. f* | wt |
| **H45** |  |  |  |  | Ranong | 2015 | *P. f* | wt |
| **H46** |  |  |  |  | Ranong | 2015 | *P. f* | wt |
| **H47** |  |  |  |  | Ranong | 2015 | *P. f* | wt |
| **H48** |  |  |  |  | Ranong | 2015 | *P. f* | C580Y |
| **H49** |  |  |  |  | Ranong | 2015 | *P. f* | wt |
| **H50** |  |  |  |  | Ranong | 2015 | *P. f* | wt |
| **H51** |  |  |  |  | Ranong | 2015 | *P. f* | wt |
| **H52** |  |  |  |  | Ranong | 2015 | *P. f* | wt |
| **H53** |  |  |  |  | Ranong | 2015 | *P. f* | wt |
| **H54** |  |  |  |  | Ranong | 2015 | *P. f* | wt |
| **H55** |  |  |  |  | Ranong | 2015 | *P. f* | wt |
| **H56** |  |  |  |  | Ranong | 2015 | *P. f* | wt |
| **H57** |  |  |  |  | Ranong | 2015 | *P. f* | C580Y |
| **H58** |  |  |  |  | Ranong | 2015 | *P. f* | C580Y |
| **H59** |  |  |  |  | Ranong | 2015 | *P. f* | C580Y |
| **H60** |  |  |  |  | Ranong | 2015 | *P. f* | C580Y |
| **H61** |  |  |  |  | Ranong | 2015 | *P. f* | C580Y |
| **H62** |  |  |  |  | Ranong | 2015 | *P. f* | wt |
| **H63** |  |  |  |  | Ranong | 2015 | *P. f* | wt |
| **H64** |  |  |  |  | Ranong | 2015 | *P. f* | wt |
| **H65** |  |  |  |  | Ranong | 2015 | *P. f* | C580Y |
| **H66** |  |  |  |  | Ranong | 2015 | *P. f* | wt |
| **H67** |  |  |  |  | Ranong | 2015 | *P. f* | wt |
| **H68** |  |  |  |  | Ranong | 2015 | *P. f* | C580Y |
| **H69** |  |  |  |  | Ranong | 2015 | *P. f* | C580Y |
| **H70** |  |  |  |  | Ranong | 2015 | *P. f* | wt |
| **H71** |  |  |  |  | Ranong | 2015 | *P. f* | wt |
| **H72** |  |  |  |  | Ranong | 2015 | *P. f* | wt |
| **H73** |  |  |  |  | Ranong | 2015 | *P. f* | C580Y |
| **H74** |  |  |  |  | Ranong | 2015 | *P. f* | C580Y |
| **H75** |  |  |  |  | Ranong | 2015 | *P. f* | wt |
| **H76** |  |  |  |  | Ranong | 2015 | *P. f* | wt |
| **H77** |  |  |  |  | Ranong | 2015 | *P. f* | wt |
| **H78** |  |  |  |  | Ranong | 2015 | *P. f* | wt |
| **H79** |  |  |  |  | Ranong | 2015 | *P. f* | wt |
| **H80** |  |  |  |  | Ranong | 2015 | *P. f* | C580Y |
| **H81** |  |  |  |  | Ranong | 2015 | *P. f* | C580Y |
| **H82** | Thai | 45 | F | Ruber plantation | Yala | 2017 | *P. f* | C580Y |
| **H83** |  |  |  |  | Ranong | 2015 | *P. f* | wt |
| **H84** |  |  |  |  | Ranong | 2015 | *P. f* | wt |
| **H85** |  |  |  |  | Ranong | 2015 | *P. f* | wt |
| **H86** |  |  |  |  | Ranong | 2015 | *P. f* | wt |
| **H87** |  |  |  |  | Ranong | 2015 | *P. f* | C580Y |
| **H88** |  |  |  |  | Ranong | 2015 | *P. f* | C580Y |
| **H89** |  |  |  |  | Ranong | 2015 | *P. f* | wt |
| **H90** |  |  |  |  | Ranong | 2015 | *P. f* | wt |
| **H91** | Thai | 6 | M | Student | Yala | 2017 | *P. f* | C580Y |

**Additional file 1. Table B.** *P. vivax* results summary by specimen.

| **ID** | Pvmdr | | Pvcrt-o | Pvdfr | | | | Pvdhfr (repeat) | Pvdhps | | | | Pvdhfr (repeat) |
| --- | --- | --- | --- | --- | --- | --- | --- | --- | --- | --- | --- | --- | --- |
|  | Y976**F** | F1076**L** | **K10** insertion | F57**L/I** | S58**R** | T61**M** | S117**T/N** | Type1(WT) VS Type2 (DELETE) | S382**A** | A83**G** | K512 **M/E** | A553**G** | Type1(WT) VS Type2 (DELETE) |
| **T1** |  |  |  |  |  |  |  |  |  |  |  |  |  |
| **T2** |  |  |  |  |  |  |  |  |  |  |  |  |  |
| **T3** |  |  |  |  |  |  |  |  |  |  |  |  |  |
| **T4** |  |  |  |  |  |  |  |  |  |  |  |  |  |
| **T5** | Y | **L** | **K10** | **I** | **R** | **M** | **T** | WT | **A** | **G** | K | **G** | WT |
| **T6** | Y | F | WT | **I** | **R** | **M** | **T** | WT | S | **G** | K | A | WT |
| **T7** | Y | F | **K10** | **I** | **R** | **M** | **T** | WT |  |  |  |  | WT |
| **T8** | **F** | **L** | **K10** | **I** | **R** | **M** | **T** | WT | **A** | **G** | **M** | **G** | WT |
| **T9** | Y | F |  |  |  |  |  |  |  |  |  |  |  |
| **T10** | **F** | **L** | K | **I** | **R** | **M** | **T** | WT |  |  |  |  | WT |
| **T11** | **F** | **L** | K | F | **R** | T | **N** | D |  |  |  |  | D |
| **T12** |  | **L** |  |  |  |  |  |  |  |  |  |  |  |
| **T13** | Y | **L** | K | **I** | **R** | **M** | **T** | WT |  |  |  |  | WT |
| **T14** | Y |  |  |  |  |  |  |  |  |  |  |  |  |
| **T15** | Y | F | K |  |  |  |  |  | **A** | **G** | K | **G** |  |
| **T16** | Y |  |  |  |  |  |  |  |  |  |  |  |  |
| **T17** | Y | **L** | K | **L** | **R** | **M** | **T** | WT | **A** | **G** | K | **G** | WT |
| **T18** | Y |  |  |  |  |  |  |  |  |  |  |  |  |
| **T19** | Y | F | K | **I** | **R** | **M** | **T** | WT | **A** | **G** | K | **G** | WT |
| **T20** | **F** | **L** | K | F | **R** | T | **N** | D | S | **G** | K | **G** | D |
| **T21** | **F** | **L** | K | **I** | **R** | **M** | **T** | WT | S | **G** | K | **G** | WT |
| **T22** | Y | F | K | **I** | **R** | **M** | **T** | WT | **A** | **G** | **M** | **G** | WT |
| **T23** |  |  |  |  |  |  |  |  |  |  |  |  |  |
| **T24** |  |  | K |  |  |  |  |  |  |  |  |  |  |
| **T25** |  |  | K | **I** | **R** | **M** | **T** | WT | **A** | **G** | K | **G** | WT |
| **T26** |  |  |  |  |  |  |  |  |  |  |  |  |  |
| **T27** |  |  | K |  |  |  |  |  |  |  |  |  |  |
| **T28** |  |  | K | **L** | **R** | **M** | **T** | WT | **A** | **G** | K | **G** | WT |
| **T29** |  |  | K | **I** | **R** | **M** | **T** | WT |  |  |  |  | WT |
| **T30** |  |  |  |  |  |  |  |  |  |  |  |  |  |
| **T31** |  |  |  |  |  |  |  |  |  |  |  |  |  |
| **T32** | Y | **L** | K | F | **R** | T | **N** | D | S | **G** | K | A | D |
| **T33** | **F** | **L** | K | **I** | **R** | **M** | **T** | WT | **A** | **G** | K | **G** | WT |
| **T34** | **F** | **L** | K | **I** | **R** | **M** | **T** | WT | A | **G** | M | **G** | WT |
| **T35** | Y | F | K | **I** | **R** | **M** | **T** | WT |  |  |  |  | WT |
| **T36** | **F** | **L** | K | **I** | **R** | **M** | **T** | WT |  |  |  |  | WT |
| **T37** | Y | F | K | **I** | **R** | **M** | **T** | WT | S | **G** | K | **A** | WT |
| **T38** |  |  | K | F | **R** | T | **N** | D |  |  |  |  | D |
| **T39** | Y | F | K | **I** | **R** | **M** | **T** | WT | S | **G** | K | **G** | WT |
| **T40** | **F** | **L** |  | F | **R** | T | **N** | D |  |  |  |  | D |
| **T41** |  |  | K | F | **R** | T | **N** | D | s | **s** | K | **A** | D |
| **T42** | Y | **L** | K | **I** | **R** | **M** | **T** | WT | **A** | **G** | K | **G** | WT |
| **T43** | Y | F |  | **I** | **R** | **M** | **T** | WT | **A** | **G** | K | **G** | WT |
| **T44** | Y | **L** | K | **I** | **R** | **M** | **T** | WT | **A** | **G** | K | **G** | WT |
| **T45** | **F** | **L** | K | F | **R** | T | **N** | D | S | **G** | K | A | D |
| **T46** |  |  |  |  |  |  |  |  |  |  |  |  |  |
| **T47** | Y | F | K | **I** | **R** | **M** | **T** | WT | S | **G** | K | **G** | WT |
| **T48** | Y | F | K | **I** | **R** | **M** | **T** | WT | A | **G** | K | **G** | WT |
| **T49** |  | F |  |  |  |  |  |  |  |  |  |  |  |
| **T50** | **F** | **L** |  | F | **R** | T | **N** | D | S | **G** | K | **A** | D |
| **T51** | **F** | **L** | K | F | **R** | T | **N** | D | S | **G** | K | **G** | D |
| **T52** |  |  | K |  |  |  |  |  | S | **G** | K | **G** |  |
| **T53** | Y | F | K | **I** | **R** | **M** | **T** | WT | **A** | **G** | K | **G** | WT |
| **T54** |  |  |  |  |  |  |  |  |  |  |  |  |  |
| **T55** | Y | F | K | F | **R** | T | **N** | D | S | **G** | K | A | D |
| **T56** | **F** | **L** |  | F | **R** | T | **N** | D |  |  |  |  | D |
| **T57** | Y | **L** |  | **I** | **R** | **M** | **T** | WT |  |  |  |  | WT |
| **T58** | **F** | **L** | K | F | **R** | T | **N** | D | S | **G** | K | **G** | D |
| **T59** | **F** | **L** |  | **I** | **R** | **M** | **T** | WT | A | **G** | K | **G** | WT |
| **T60** | Y | F |  | F | **R** | T | **N** | D | S | **G** | K | **G** | D |
| **T61** | Y | F | K | **I** | **R** | **M** | **T** | WT |  |  |  |  | WT |
| **T62** | Y | **L** |  | **I** | **R** | **M** | **T** | WT | **A** | **G** | K | **G** | WT |
| **T63** | **F** | **L** |  | **I** | **R** | **M** | **T** | WT |  |  |  |  | WT |
| **T64** | **F** | F |  | **I** | **R** | **M** | **T** | WT | **A** | **G** | K | G | WT |
| **T65** | Y | F |  | **I** | **R** | **M** | **T** | WT | **A** | **G** | K | A | WT |
| **T66** | **F** | **L** |  | **I** | **R** | **M** | **T** | WT | S | **G** | K | A | WT |
| **T67** | Y | **L** |  | **I** | **R** | **M** | **T** | WT | S | **G** | K | **G** | WT |
| **T68** | Y | **L** |  |  |  |  |  |  | S | **G** | K | **G** |  |
| **T69** |  | **L** |  |  |  |  |  |  |  |  |  |  |  |
| **T70** |  | **L** |  |  |  |  |  |  |  |  |  |  |  |
| **T71** |  | **L** |  |  |  |  |  |  |  |  |  |  |  |
| **T72** |  | **L** |  |  |  |  |  |  |  |  |  |  |  |
| **T73** |  | **L** |  |  |  |  |  |  |  |  |  |  |  |
| **T74** |  | **L** |  |  |  |  |  |  |  |  |  |  |  |
| **T75** | **F** | **L** |  |  |  |  |  |  | S | **G** | K | G |  |
| **T76** | **F** | **L** |  | **I** | **R** | **M** | **T** | WT | **S** | **G** | K | **G** | WT |
| **T77** |  |  |  | **I** | **R** | **M** | **T** | WT |  |  |  |  | WT |
| **T78** |  |  |  | **I** | **R** | **M** | **T** | WT |  |  |  |  | WT |
| **T79** |  |  |  |  |  |  |  | WT |  |  |  |  | WT |
| **T80** |  |  |  |  |  |  |  | WT |  |  |  |  | WT |
| **T81** |  |  |  | **I** | **R** | **M** | **T** | WT | **S** | **G** | **K** | **G** | WT |
| **T82** |  |  |  | **I** | **R** | **M** | **T** | WT |  |  |  |  | WT |
| **T83** |  |  |  |  |  |  |  |  |  |  |  |  |  |
| **T84** |  |  |  |  |  |  |  |  |  |  |  |  |  |
| **T85** |  |  |  |  |  |  |  |  |  |  |  |  |  |
| **T86** |  |  |  |  |  |  |  |  |  |  |  |  |  |
| **T87** |  |  |  |  |  |  |  |  |  |  |  |  |  |
| **T88** |  |  |  |  |  |  |  |  |  |  |  |  |  |
| **T89** |  |  |  |  |  |  |  |  |  |  |  |  |  |
| **T90** |  |  |  |  |  |  |  |  |  |  |  |  |  |
| **T91** |  |  |  |  |  |  |  |  |  |  |  |  |  |
| **T92** |  |  |  |  |  |  |  |  |  |  |  |  |  |
| **T93** |  |  |  |  |  |  |  |  |  |  |  |  |  |
| **T94** | Y | **L** | WT | **L** | **R** | **M** | **T** | WT | **S** | **A** | **K** | **A** | WT |
| **T95** | Y | **L** | WT | **L** | **R** | **M** | **T** | WT | **S** | **A** | **K** | **A** | WT |
| **T96** | Y | **L** |  |  |  |  |  | WT | **S** | **A** | **K** | **A** |  |
| **T97** | Y | **L** | K | **L** | **R** | **M** | **T** | WT | **S** | **A** | **K** | **A** | WT |
| **T98** | Y | **L** |  |  |  |  |  |  | **S** | **A** | **K** | **A** |  |
| **T99** | Y | **L** |  | **L** | **R** | **M** | **T** | WT | **S** | **A** | **K** | **A** | WT |
| **T100** | Y | **L** |  |  |  |  |  |  |  |  |  |  |  |
| **T101** | Y | **L** | K | **L** | **R** | **M** | **T** | WT |  |  |  |  | WT |
| **T102** | Y | **L** | K | **L** | **R** | **M** | **T** | WT |  |  |  |  | WT |
| **T103** | Y | **L** | K | **L** | **R** | **M** | **T** | WT |  |  |  |  | WT |
| **T104** | Y | **L** | K | **L** | **R** | **M** | **T** | WT |  |  |  |  | WT |
| **T105** | Y | **L** | K | **L** | **R** | **M** | **T** | WT |  |  |  |  | WT |
| **T106** | Y | **L** | K | **L** | **R** | **M** | **T** | WT |  |  |  |  | WT |
| **T107** | Y | **L** | K | **L** | **R** | **M** | **T** | WT |  |  |  |  | WT |
| **T108** | Y | **L** |  | **L** | **R** | **M** | **T** | WT |  |  |  |  | WT |
| **T109** |  |  |  | **L** | **R** | **M** | **T** | WT | **S** | **A** | **K** | **A** | WT |
| **T110** |  |  |  | **L** | **R** | **M** | **T** | WT |  |  |  |  | WT |
| **T111** |  |  |  | **L** | **R** | **M** | **T** | WT |  |  |  |  | WT |
| **T112** | Y | **L** | K | **L** | **R** | **M** | **T** | WT |  |  |  |  | WT |
| **T113** | Y | **L** |  | **L** | **R** | **M** | **T** | WT | **S** | **A** | **K** | **A** | WT |
| **T114** | Y | **L** |  | **L** | **R** | **M** | **T** | WT | **S** | **A** | **K** | **A** | WT |
| **T115** | Y | **L** |  | **L** | **R** | **M** | **T** | WT |  |  |  |  | WT |
| **T116** | Y | **L** | K | **L** | **R** | **M** | **T** | WT |  |  |  |  | WT |
| **T117** | Y | **L** |  | **L** | **R** | **M** | **T** | WT | **S** | **A** | **K** | **A** | WT |
| **T118** | Y | **L** |  | **L** | **R** | **M** | **T** | WT | S | A | K | A | WT |
| **T119** | Y | **L** | K | **L** | **R** | **M** | **T** | WT | S | A | K | A | WT |
| **T120** | Y | **L** |  | **L** | **R** | **M** | **T** | WT |  |  |  |  | WT |
| **T121** | Y | **L** |  | **L** | **R** | **M** | **T** | WT | S | A | K | A | WT |
| **T122** | Y | **L** |  | **L** | **R** | **M** | **T** | WT | S | A | K | A | WT |
| **T123** | Y | **L** |  | **L** | **R** | **M** | **T** | WT | S | A | K | A | WT |
| **T124** | Y | **L** |  | **L** | **R** | **M** | **T** | WT |  |  |  |  | WT |
| **T125** | Y | **L** |  | **L** | **R** | **M** | **T** | WT | S | A | K | A | WT |
| **T126** | Y | **L** |  | **L** | **R** | **M** | **T** | WT | S | A | K | A | WT |
| **T127** | Y | **L** |  | **L** | **R** | **M** | **T** | WT |  |  |  |  | WT |
| **T128** | Y | **L** |  | **L** | **R** | **M** | **T** | WT |  |  |  |  | WT |
| **T129** | Y | **L** |  | **L** | **R** | **M** | **T** | WT | S | A | K | A | WT |
| **T130** | Y | **L** |  | **L** | **R** | **M** | **T** | WT |  |  |  |  | WT |
| **T131** | Y | **L** |  | **L** | **R** | **M** | **T** | WT |  |  |  |  | WT |
| **T132** | Y | **L** |  | **L** | **R** | **M** | **T** | WT | S | A | K | A | WT |
| **T133** |  |  |  | **L** | **R** | **M** | **T** | WT |  |  |  |  | WT |
| **T134** | Y | **L** |  | **L** | **R** | **M** | **T** | WT |  |  |  |  | WT |
| **T135** | Y | **L** |  | **L** | **R** | **M** | **T** | WT | S | A | K | A | WT |
| **T136** | Y | **L** |  | **L** | **R** | **M** | **T** | WT |  |  |  |  | WT |
| **T137** | Y | **L** |  | **L** | **R** | **M** | **T** | WT | S | A | K | A | WT |
| **T138** | Y | **L** | K | **L** | **R** | **M** | **T** | WT |  |  |  |  | WT |
| **T139** |  |  |  | **L** | **R** | **M** | **T** | WT |  |  |  |  | WT |
| **T140** | Y | **L** | K | **L** | **R** | **M** | **T** | WT |  |  |  |  | WT |
| **T141** | Y | **L** |  | **L** | **R** | **M** | **T** | WT | S | A | K | A | WT |
| **T142** | Y | **L** |  | **L** | **R** | **M** | **T** | WT |  |  |  |  | WT |
| **T143** | Y | **L** |  | **L** | **R** | **M** | **T** | WT |  |  |  |  | WT |
| **T144** |  |  |  | **L** | **R** | **M** | **T** | WT | S | A | K | A | WT |
| **T145** | Y | **L** |  | **L** | **R** | **M** | **T** | WT |  |  |  |  | WT |
| **T146** | Y | **L** |  |  |  |  |  |  |  |  |  |  |  |
| **T147** | Y | **L** |  | **L** | **R** | **M** | **T** | WT |  |  |  |  | WT |
| **T148** | Y | **L** | WT |  |  |  |  |  |  |  |  |  |  |
| **T149** | Y | **L** | WT | **L** | **R** | **M** | **T** | WT | S | A | K | A | WT |
| **T150** |  |  |  | **L** | **R** | **M** | **T** | WT | S | A | K | A | WT |
| **T151** | Y | **L** | WT | **L** | **R** | **M** | **T** | WT | S | A | K | A | WT |
| **T152** | Y | **L** | WT | **L** | **R** | **M** | **T** | WT | S | A | K | A | WT |
| **T153** | Y | **L** | K | **L** | **R** | **M** | **T** | WT | S | A | K | A | WT |
| **T154** | Y | **L** | K | **L** | **R** | **M** | **T** | WT |  |  |  |  | WT |
| **T155** | Y | **L** | K | **L** | **R** | **M** | **T** | WT |  |  |  |  | WT |
| **T156** | Y | **L** | K | F | S | T | **S** | D | S | A | K | A | D |
| **T157** |  |  |  |  |  |  |  |  |  |  |  |  |  |
| **T158** | Y | **L** | K | **L** | **R** | **M** | **T** | WT | S | A | K | A | WT |
| **T159** | Y | **L** |  | **L** | **R** | **M** | **T** | WT |  |  |  |  | WT |
| **T160** | Y | **L** | K | **L** | **R** | **M** | **T** | WT | S | A |  |  | WT |
| **T161** | Y | **L** |  | **L** | **R** | **M** | **T** | WT | S | A | K | A | WT |
| **T162** | Y | **L** |  | **L** | **R** | **M** | **T** | WT | S | A |  |  | WT |
| **T163** |  |  |  | **L** | **R** | **M** | **T** | WT | S | A | K | A | WT |
| **T164** | Y | **L** |  | **L** | **R** | **M** | **T** | WT | S | A | K | A | WT |
| **T165** | Y | **L** |  | **L** | **R** | **M** | **T** | WT |  |  |  |  | WT |
| **T166** | Y | **L** |  |  |  |  |  |  | S | A | K | A |  |
| **T167** |  |  |  | **L** | **R** | **M** | **T** | WT | S | A | K | A | WT |
| **T168** | Y | **L** | K |  |  |  |  | WT |  |  |  |  | WT |
| **T169** | Y | **L** |  | **I** | **R** | **M** | **T** |  |  |  |  |  |  |
| **T170** | Y | **L** |  | **L** | **R** | **M** | **T** | WT | S | A | K | A | WT |
| **T171** | Y | **L** |  | **L** | **R** | **M** | **T** | WT | S | A | K | A | WT |
| **T172** |  |  |  | **L** | **R** | **M** | **T** | WT | S | A | K | A | WT |
| **T173** | Y | **L** | K | **L** | **R** | **M** | **T** | WT | S | A | K | A | WT |
| **T174** | Y | **L** | K | **L** | **R** | **M** | **T** | WT | S | A | K | A | WT |
| **T175** | Y | **L** |  | **L** | **R** | **M** | **T** | WT | S | A | K | A | WT |
| **T176** | Y | **L** | K | **L** | **R** | **M** | **T** | WT | S | A | K | A | WT |
| **T177** |  |  |  | **L** | **R** | **M** | **T** | WT |  |  |  |  | WT |
| **T178** |  |  |  | **L** | **R** | **M** | **T** | WT |  |  |  |  | WT |
| **T179** |  |  |  | **L** | **R** | **M** | **T** | WT |  |  |  |  | WT |
| **T180** |  |  |  | **L** | **R** | **M** | **T** | WT |  |  |  |  | WT |
| **T181** | Y | **L** | K | **L** | **R** | **M** | **T** | WT |  |  |  |  | WT |
| **T182** |  |  |  | **F** | **R** | T | **N** | D |  |  |  |  | D |
| **T183** |  |  |  | **L** | **R** | **M** | **T** | WT |  |  |  |  | WT |
| **T184** |  |  |  | **L** | **R** | **M** | **T** | WT |  |  |  |  | WT |
| **T185** |  |  |  | **L** | **R** | **M** | **T** | WT |  |  |  |  | WT |
| **T186** |  |  |  | **L** | **R** | **M** | **T** | WT |  |  |  |  | WT |
| **T187** | Y | **L** | K | **L** | **R** | **M** | **T** | WT |  |  |  |  | WT |
| **T188** | Y | **L** | K | **L** | **R** | **M** | **T** | WT |  |  |  |  | WT |
| **T189** | Y | **L** | K | **L** | **R** | **M** | **T** | WT |  |  |  |  | WT |
| **T190** | Y | **L** | K | **L** | **R** | **M** | **T** | WT |  |  |  |  | WT |
| **T191** | Y | **L** | K | **L** | **R** | **M** | **T** | WT |  |  |  |  | WT |
| **T192** |  |  |  | **L** | **R** | **M** | **T** | WT |  |  |  |  | WT |
| **T193** |  |  |  | **L** | **R** | **M** | **T** | WT |  |  |  |  | WT |
| **T194** |  |  |  | **L** | **R** | **M** | **T** | WT |  |  |  |  | WT |
| **T195** | Y | **L** |  | **L** | **R** | **M** | **T** | WT |  |  |  |  | WT |
| **T196** | Y | **L** | K | **L** | **R** | **M** | **T** | WT |  |  |  |  | WT |
| **T197** | Y | **L** |  | **L** | **R** | **M** | **T** | WT |  |  |  |  | WT |
