# Supplementary material for: Molecular detection of drug resistant malaria in Southern Thailand
Source: Malar J. 2019 Aug 15;18:275. doi: 10.1186/s12936-019-2903-y (PMC6694568; doi:10.1186/s12936-019-2903-y)
Supplement: Supplementary file 2 — Additional file 2: Table S2. PCR primers used in study. [file 12936_2019_2903_MOESM2_ESM.docx]

**Additional file 2: Table S2.** PCR Primers used in study.

| Name | Sequences 5’-3’ | *Gene targets* | *Product size (bp)* | *References* |
| --- | --- | --- | --- | --- |
| Pvdhfr (outer) F  Pvdhfr (outer) R | CACCGCACCAGTTGATTCCT  CCTCGGCGTTGTTCTTCT | *Pvdhfr* | 979 | (25) |
| Pvdhfr (nested) F  Pvdhfr (nested) R | CCCCACCACATAACGAAG  CCCCACCTTGCTGTAAACC | *Pvdhfr* | 755 | (25) |
| Pvdhps (outer) F  Pvdhps (outer) R | GATGGCGGTTTATTTGTCG  GCTGATCTTTGTCTTGACG | *Pvdhps* | 1009 | (25) |
| Pvdhps (nested) F  Pvdhps (nested) R | GCTGTGGAGAGGATGTTC  CCGCTCATCAGTCTGCAC | *Pvdhps* | 731 | (25) |
| Pvcrt-o F  Pvcrt-o R | CAGTGAGAAGCCCCTGTTCG  CCGCTCATCAGTCTGCAC | *Pvcrt-o* | 750 | ~ |
| Pvmdr F  Pvmdr R | GCGAACTCGAATAAGTACTCCCTCTA  GGCGTAGCTTCC CGTAAATAAA | *Pvmdr1* | 762 | (26) |
| Pfk13 F (outer)  Pfk13 R (outer) | GCCTTGTTGAAAGAAGCAGAA  CGCCATTTTCTCCTCCTGTA | *Pfkelch13* | 792 | (24). |
| Pfk13 F (nested)  Pfk13 R (nested) | GCCTTGTTGAAAGAAGCAGAA GTGGCAGCTCCAAAATTCAT | *Pfkelch13* | 747 | (24). |
